# Innovative Approaches to Obtain Minors’ Consent for Biomedical HIV Prevention Trials: Multi-Site Quasi-Experimental Study of Adolescent and Parent Perspectives

**DOI:** 10.2196/16509

**Published:** 2020-03-30

**Authors:** Amelia Knopf, Mary A Ott, Claire Burke Draucker, J Dennis Fortenberry, Daniel H Reirden, Renata Arrington-Sanders, John Schneider, Diane Straub, Rebecca Baker, Giorgos Bakoyannis, Gregory D Zimet

**Affiliations:** 1 Department of Community & Health Services School of Nursing Indiana University Indianapolis, IN United States; 2 Department of Pediatrics School of Medicine Indiana University Indianapolis, IN United States; 3 Children's Hospital Colorado School of Medicine The University of Colorado Aurora, CO United States; 4 Division of General Pediatrics & Adolescent Medicine School of Medicine Johns Hopkins University Baltimore, MD United States; 5 Department of Medicine The University of Chicago Chicago, IL United States; 6 Morsani College of Medicine University of South Florida Tampa, FL United States; 7 Department of Biostatistics School of Medicine Indiana University Indianapolis, IN United States

**Keywords:** HIV, biomedical ethics, adolescence, parental consent

## Abstract

**Background:**

Despite the high burden of new HIV infections in minor adolescents, they are often excluded from biomedical HIV prevention trials, largely owing to the ethical complexities of obtaining consent for enrollment. Researchers and ethics regulators have a duty to protect adolescents—as a special category of human subjects, they must have protection that extends beyond those afforded to all human subjects. Typically, additional protection includes parental consent for enrollment. However, parental consent can present a risk of harm for minor adolescents. Research involving minor adolescents indicate that they are unwilling to join biomedical trials for stigmatized health problems, such as HIV, when parental consent is required. This presents a significant barrier to progress in adolescent HIV prevention by creating delays in research and the translation of new scientific evidence generated in biomedical trials in adult populations.

**Objective:**

This protocol aims to examine how parental involvement in the consent process affects the acceptability of hypothetical participation in biomedical HIV prevention trials from the perspectives of minor adolescents and parents of minor adolescents.

**Methods:**

In this protocol, we use a quasi-experimental design that involves a simulated consent process for 2 different HIV prevention trials. The first trial is modeled after an open-label study of the use of tenofovir disoproxil fumarate and emtricitabine as preexposure prophylaxis for HIV. The second trial is modeled after a phase IIa trial of an injectable HIV integrase inhibitor. There are 2 groups in the study—minor adolescents aged 14 to 17 years, inclusive, and parents of minor adolescents in the same age range. The adolescent participants are randomized to 1 of 3 consent conditions with varying degrees of parental involvement. After undergoing a simulated consent process, they rate their willingness to participate (WTP) in each of the 2 trials if offered the opportunity. The primary outcome is WTP, given the consent condition. Parents undergo a similar process but are asked to rate the acceptability of each of the 3 consent conditions. The primary outcome is acceptability of the consent method for enrollment. The secondary outcomes include the following: capacity to consent among both participant groups, the prevalence of medical mistrust, and the effects of the study phase (eg, phase IIa vs the open-label study) and drug administration route (eg, oral vs injection) on WTP (adolescents) and acceptability (parents) of the consent method.

**Results:**

Enrollment began in April 2018 and ended mid-September 2019. Data are being analyzed and dissemination is expected in April 2020.

**Conclusions:**

The study will provide the needed empirical data about minor adolescents’ and parents’ perspectives on consent methods for minors. The evidence generated can be used to guide investigators and ethics regulators in the design of consent processes for biomedical HIV prevention trials.

**International Registered Report Identifier (IRRID):**

DERR1-10.2196/16509

## Introduction

### Background

Minor adolescents (those aged younger than 18 years) and young adults (aged 18-24 years) account for more than 1 in 5 new HIV infections in the United States. Sexual and gender minorities make up 80% of incident infections in adolescents and young adults, and African American men and transgender women account for 80% of infections among the sexual and gender minority youth [1]. Across age groups, minor adolescents are the least likely to know they have HIV, be connected to HIV care, and be virally suppressed [1]. Other at-risk populations have experienced significant declines in HIV rates with access to biomedical prevention interventions such as preexposure prophylaxis (PrEP), but similar declines have not been observed in minor adolescents [2]. PrEP, which involves taking antiretroviral medication, is up to 95% effective in preventing HIV acquisition. The first PrEP regimen, once daily oral tenofovir disoproxil fumarate and emtricitabine (TDF-FTC), was approved for use in adults at risk of HIV in 2012. Labeling for use with at-risk minor adolescents (those aged younger than 18 years) was delayed by 6 years owing to scarce data on the safety and tolerability of TDF-FTC as PrEP for minors [3].

Minor adolescents are often excluded from biomedical research. A recent analysis of 388 phase III and phase IV National Institutes of Health (NIH)–funded trials indicated minor adolescents and children were excluded from 75% of the studies [4]. In biomedical HIV prevention, less than 1% of clinical trials included minor adolescents [5,6]. The exclusion of minors and other vulnerable populations from clinical trials impedes the translation of science to public health practice, as is evident in the 6-year lag between the approval of TDF-FTC as PrEP for adults and the labeling indication for use with minors.

Investigators are often reluctant to engage minor adolescents in biomedical research on stigmatized conditions owing to the ethical complexities involved [5-7]. Minors are considered a vulnerable research population, and there are additional regulatory requirements for research with minors that extend beyond those afforded to all research participants [8-10]. The cornerstone of these additional protections for minors has been parental involvement in the consent process [8]. In this assent/permission model, a minor must assent to participation in the study, and their parent or guardian must provide permission for the minor to enroll.

Researchers have examined the negotiation of minor assent and parental permission within the context of therapeutic trials for chronic illnesses such as asthma and cancer. In this context, roughly 60% to 70% of adolescent-parent dyads agree on the enrollment decision [11,12]. Recent research suggests a similar concordance (56%) among adolescent-parent dyads who were asked to consider a hypothetical scenario in which the minor adolescent would enroll in a biomedical HIV prevention study [13,14]. The same research team found that 73% of discordant dyads were able to resolve their discordance through communication about their perspectives, often in a short time (median 2.5 min) [14].

Although the assent/permission model works reasonably well for enrolling minors in nonstigmatized research, it fails for research that poses greater than minimal risk of harm and addresses a stigmatized health condition or targets a socially marginalized population [15-18]. Parental engagement in the consent process introduces the risk of social harm, as research participation may result in the disclosure of sexual behaviors, sexual orientation, or gender identity. This type of disclosure, particularly for sexual and gender minority adolescents, can result in physically or psychologically abusive responses from parents or the minor being kicked out of their home. Exploratory work with minor adolescents whose sexual behaviors or demographic characteristics are indicative of a risk of HIV acquisition indicates that they are unwilling to risk social harm to enroll in biomedical research [19,20].

Research on decision making and cognition indicates that minor adolescents are reliably capable of understanding research concepts and providing informed consent [21,22]. These works argue for the consideration of the ways in which adolescents are vulnerable, rather than using age alone as a proxy for vulnerability [21,22]. Minors’ capacity to consent is recognized in the willingness of institutional review boards (IRBs) to waive parental permission and allow adolescent self-consent for behavioral studies. For biomedical HIV prevention, regulatory concerns have been specifically addressed by the Food and Drug Administration, which states that minor self-consent for biomedical HIV prevention research is permissible under federal regulations [9,23]. However, our own work demonstrates that researchers and IRB members are concerned about minor self-consent, and many see parental permission as a way to protect minors, support parent rights, and decrease liability for institutions [24,25].

Missing in the discussion are data from the youth and parents on acceptability and preferences around consent to biomedical HIV prevention trials. It is especially critical to understand the role of participants’ racial and ethnic identity, their sexual orientation and disclosure to parents, and features of the biomedical HIV prevention drug or device as well as its stage of development. Black Americans experience structural and individual barriers to accessing health care [26], and their history of unequal treatment in both clinical care and research settings has resulted in the mistrust of health care providers and researchers. This mistrust is evident in research with young black sexual and gender minorities, who describe not only a general mistrust of research and pharmaceutical companies but also a specific concern that HIV prevention interventions could be designed to infect them with HIV rather than prevent infection [27,28]. Black parents who participated in a study of parental perspectives on minor participation in biomedical HIV prevention research also mentioned that the historic mistreatment of black research participants may affect black parents’ willingness to allow their minors to enroll in research [29].

Recent research has indicated *outness* (the degree to which a sexual minority adolescent has disclosed their sexual orientation to parents, family members, and friends) has a significant association with willingness to participate (WTP) in HIV research. For example, Nelson et al [30] report that minors who were not out to guardians had 5 times greater odds of saying they would not participate in a future HIV study than those who were out to guardians. Mustanski et al [31] report similar associations between outness and WTP in HIV prevention trials.

Recently, 2 biomedical HIV prevention studies have included minor adolescents. The first, Adolescent Medicine Trials Network for HIV/AIDS Interventions protocol 113 (ATN 113), enrolled minor adolescents aged 15 to 17 years in an open-label study of the safety of and adherence to TDF-FTC as HIV PrEP. The second, Microbicide Trials Network (MTN) 023, enrolled adolescents aged 15 to 17 years in a phase IIa trial of the safety of and adherence to a dapivirine (25 mg) vaginal ring. These two trials differed in several important ways. First, the approaches to consent were different—participants in ATN 113 were permitted to self-consent, whereas MTN 023 required participants to have parental consent. Second, the drug delivery mechanisms were different—a tablet taken by mouth (TDF-FTC, ATN 113) vs a vaginal ring delivery system (dapivirine 25 mg, MTN 023). Finally, the stages of drug development and testing were different—the ATN 113 study was an open-label study of a drug with a well-known safety and tolerability profile owing to its long history of use in HIV treatment. Conversely, the MTN 023 study was a phase IIa study of an investigational new drug. Although the approaches to minor consent were different, to the best of our knowledge, there are no published studies that explore the effects of the drug delivery mechanism and the stage of drug development on (1) minors’ WTP in biomedical HIV prevention trials or (2) parents’ perspectives on the acceptability of minor consent. We add to the body of work on minor consent to participation in biomedical HIV prevention trials by examining the relationship between participant characteristics and features of the HIV prevention intervention as well as its stage of development.

### Objective

Here, we describe the protocol for our ongoing study researching consent for minors. The project is titled *Consent 2.0* and is supported by the NIH-funded Adolescent Medicine Trials Network for HIV/AIDS Interventions (ATN). *Consent 2.0* examines the issue of minor consent to enrollment in biomedical HIV prevention trials from the perspective of the youth and parents. The study will expand the body of empirical evidence available to guide regulators, IRB members, researchers, and policy makers as they consider approaches to the ethical engagement of minor adolescents in biomedical research on stigmatized topics or with highly vulnerable populations. The purpose of the study is to examine how parental involvement in the consent process affects the acceptability of hypothetical participation in biomedical HIV prevention trials, from the perspective of minor adolescents and parents of minor adolescents. We use a simulated consent process, which emulates real consent processes for 2 different types of biomedical HIV prevention trials that have included minor adolescents.

With both adolescent and parent participants, we examine the effects of 3 possible consent processes: condition 1: minor self-consent; condition 2: adult permission required, with an option to select either a parent/guardian or a study-appointed ombudsperson; and condition 3: parental permission required. Under condition 1, the minor adolescent can consent to enrollment without seeking parental permission. Under condition 2, the minor adolescent is required to obtain permission to enroll from an adult and may choose between a parent/guardian or an ombudsperson. The ombudsperson is an adult who is familiar with the study and its risks and benefits and helps the adolescent arrive at the best decision for themselves. Under condition 3, the minor is required to have parental permission to enroll and assent to enrollment.

The study has 3 aims. First, we aim to describe how consent conditions influence minor adolescents’ WTP in biomedical HIV prevention trials. In particular, we will evaluate if WTP in biomedical trials is affected by adolescents’ concern about HIV, demographic characteristics (eg, race, ethnicity, and sexual and gender identity), family context, and medical mistrust. The second aim is to describe parents’ attitudes toward the various consent models, their opinions of the risks and benefits of each model, and their conceptualization of a shared decision-making process for consent. Finally, the third aim is to describe the effects of the study agent’s stage of development (eg, a drug with a well-established safety profile vs a new drug with an unknown safety profile) and the mechanism of delivery (eg, oral vs injection or intravaginal delivery) on minors’ WTP and parents’ perspectives on the acceptability of the different consent conditions.

## Methods

### Study Design Overview

We are using a quasi-experimental design to explore how the informed consent process affects the acceptability of biomedical HIV prevention trials from the perspective of sexually active minor adolescents and the parents of minor adolescents. All study participants complete a computer-assisted self-interview (CASI) that collects demographic, social, behavioral, and attitudinal measures. Then, participants undergo a simulated consent process for 2 different studies. The first study is modeled after the ATN 113, an open-label study of oral TDF-FTC as PrEP. The second study is modeled after the HIV Prevention Trials Network protocol 077 (HPTN 077), a phase IIa trial of an injectable HIV integrase inhibitor that is preceded by an oral lead-in of the same drug. Participants answer questions about the 2 studies.

### Study Setting

We are recruiting participants from 4 partnering research sites in the following US cities: Baltimore, Maryland (partnering organization: Johns Hopkins University), Chicago, Illinois, (partnering organization: the University of Chicago), Aurora, Colorado (partnering organization: the University of Colorado School of Medicine), and Tampa, Florida (partnering organization: the University of South Florida). These cities have diverse populations, high rates of incident HIV infection among adolescents and young adults, and demonstrated success in recruiting minor adolescents for biomedical HIV research. Our partnering organizations have a history of recruiting sexual and gender minority adolescents of color for HIV research, which is also a strength of our selected sites.

### Study Population

We are recruiting adolescents between the ages of 14 and 17 years, inclusive, who are able to read and speak English, are either HIV negative or uncertain of their HIV status, and have engaged in at least one sexual behavior associated with an increased risk of HIV (see Textboxes 1 and 2) in the last 12 months. The study is also recruiting adults who are able to read and speak English and are currently parenting an adolescent between the ages of 14 and 17 years, inclusive, whose HIV status is either negative or unknown. Adult participants are not parents or guardians of youth participants. All potentially eligible participants are asked to provide partial addresses that are assessed for matched pairs; any participant whose partial address matches that of a previously enrolled participant is rendered ineligible to participate in the study. Recruitment efforts begin with on-site outreach within the adolescent medicine clinics affiliated with our research sites. Our research sites were chosen for their diverse patient populations, their location in urban areas with ongoing HIV clinical trials, and their history of service to sexual and gender minority youth. Site-based recruitment efforts are supplemented by further recruitment via social media advertising, printed fliers, and word of mouth. Social media advertisements are designed with support from a racially and ethnically diverse group of adolescents and young adults from 2 of our partnering research sites.

**Sexual behavior inclusion criteria, by the sex assigned at birth—male. The adolescent must indicate engagement in at least one of the following behaviors to be considered eligible.**

During the last 12 months, which of the following is true for you? (check all that apply):

- I had unprotected anal sex with a male
- I had protected anal sex with 3 or more males
- I had sex with a male for money, gifts, shelter, or drugs
- I had sex with a male, and I have had a sexually transmitted infection (gonorrhea, chlamydia, syphilis, herpes)
- I had sex with someone who is HIV+
- I had anal sex with a male and the condom slipped off or broke

**Sexual behavior inclusion criteria, by the sex assigned at birth—female. The adolescent must indicate engagement in at least one of the following behaviors to be considered eligible.**

During the last 12 months, which of the following is true for you? (check all that apply):

- I had unprotected anal or vaginal sex with a male
- I had sex with someone who is HIV+
- I had protected vaginal or anal sex with 3 or more males
- I had sex with a male for money, gifts, shelter, or drugs
- I have had sex with one or more males, and I have had a sexually transmitted infection (gonorrhea, chlamydia, syphilis, herpes)
- I had vaginal or anal sex with a male and the condom slipped off or broke

### Randomization

Adolescents are being randomized in a 1:1:1 ratio into 1 of 3 consent conditions (see the Study Visit section) using block randomization with a block size of k=3 (in every 3 subjects, exactly 1 is allocated to each condition). The randomization is stratified by study site and sex assigned at birth. Within the study site, the sex assigned at birth, and consent condition, the order in which the hypothetical trials are presented to adolescents is block randomized with a block size of k=2.

Parents indicate the acceptability of each of the 3 consent conditions for each of the 2 hypothetical trials. Independent of the sex strata, the order of hypothetical trial presentation is block randomized with a block size of k=2. Separately for each hypothetical trial, the order of evaluation for the 3 consent conditions is randomized so that each ordering is equally likely.

### Compensation

All participants receive US $50, in cash or gift cards, to compensate for their time. An additional US $25 is provided to participants who complete the debriefing interview. Each site determines the most appropriate form of compensation. In addition, sites offer reimbursement for transportation. Each site determines the most appropriate form of transportation reimbursement (eg, bus fare, subway tokens, taxi vouchers, or cash). Participants who make a separate or unnecessary trip to the study site for screening but are deemed ineligible for any reason—including the lack of interest—receive US $10 (cash or gift cards) as well as US $5 transportation reimbursement (eg, bus pass).

### Study Visit

All participants will be asked to complete the interview until we meet our aim of 48-64 interviews—6-8 adolescents and 6-8 parents from each of the 4 study sites At the initiation of the visit, participants review the Consent 2.0 study information sheet with a research assistant and verbally consent to proceed with the study. The study visit has 2 key elements—simulated consent procedures for the 2 clinical trials and a CASI. As previously mentioned, a subset of participants complete a debriefing interview.

### Simulated Consent Procedures

We adapted the consent forms from 2 existing biomedical HIV prevention trials. These 2 trials differed by route of administration, phase of the trial, and if the study drug was already approved as PrEP for adults. The first was an open-label study of the safety of and tolerability to oral TDF-FTC as PrEP for minors (ATN 113), and the second was a phase IIa randomized controlled trial of cabotegravir as PrEP, delivered via an oral lead-in followed by a long-acting injection (HPTN 077). All participants undergo simulated consent procedures for both trials. As noted above, to prevent ordering effects on the outcomes of interest, half of our participants begin with the ATN 113 consent, and the other half begin with the HPTN 077 consent. For each, participants read study summaries on their own and then have a consent conversation with a research assistant who talks to the participant as though they are actually preparing to enroll in the trial.

#### Adolescents

At enrollment in our study, adolescents are randomized to 1 of the 3 consent conditions (minor self-consent, adult permission required with the option to select either a parent/guardian or a study-appointed ombudsperson, and parental permission required). Their consent condition is emphasized at the end of the simulated consent procedure. For example, if assigned to condition 3 (parental permission required), the research assistant will conclude the consent conversation saying, “Now we’ve come to the point at which you would decide if you want to join this study. If you did want to join the study, you would need to ask your parent or guardian to give your permission to join.” Next, the adolescent answers a series of questions via a CASI, including the primary outcome question, *If offered the chance, how likely would you be to participate in the study you just heard about?* which is a Likert-type question with a range of 1 to 5, where 1 is *definitely would not participate,* and 5 is *definitely would participate*. After the participant answers the CASI questions, the research assistant asks a series of questions focused on the understanding of the study, adapted from the University of California, San Diego Brief Assessment of Capacity to Consent (UBACC) [32]. The process is then repeated for the second trial.

#### Parents

Parents undergo the simulated consent procedures as described above. However, they are not randomized to a consent condition. Instead, they answer a Likert-type question about the acceptability of each consent condition, which is described in a brief vignette. For example, the vignette for consent condition 2, adult permission required, is as follows:

Imagine your teen wants to join the study we just described. Your teen comes to the research clinic on their own. They read the consent form and have an opportunity to ask questions. Your teen is required to have an adult’s permission to sign up for the study. They can choose to ask either you or a neutral adult, called an “ombudsman.” The ombudsman is not in charge of the study; the ombudsman’s job is to ensure your teen understands the research study and to help your teen think about the risks and benefits of joining the study. Your teen would need either your permission OR the ombudsman’s permission to join the study.

In this approach to consent, your teen must have an adult’s permission to join the study; your teen would be able to choose whether to seek permission from you or the ombudsman.

After reading the scenario, the parent rates the acceptability of this approach to research consent on a scale of 1 to 5, with 1 being *completely unacceptable* and 5 being *completely acceptable*. These vignettes are presented in a random order to prevent ordering effects. The research assistant asks the series of questions focused on the understanding of the study, and then the entire process is repeated for the second trial.

### Computer-Assisted Self-Interview Questions

All participants answer demographic, behavioral, and attitudinal questions via a CASI. A brief description of measures can be found in the following section.

### Debriefing Interviews

At the end of the study visit, participants are asked if they would like to stay for a debriefing interview to further explain their perspectives on consent and HIV prevention trials. All participants will be asked to complete the interview until we have at least eight adolescent and eight parent participants from each site. If we find a substantive variation in the interviews, we will continue interviewing participants. For adolescents, the questions include: *Tell me about your parents*, *What is your relationship like with them?* and *What are your thoughts on medical research?* For parents, questions focus on the teen (eg, *Have you talked to your teen about their sexual orientation?* *Tell me about that conversation*, and *Is it ever okay for teens to self-consent?*

### Sample Size and Power Calculation

This study will enroll approximately 144 (36 per study site) minor adolescents and 120 (30 per study site) parents. On the basis of a linear regression model with consent condition as the independent categorical variable, we calculated that we will need 120 participants (40 per consent condition) to achieve an 80% power for detecting an effect size (f^2^) of 0.084, which is between a small (f^2^=0.02) and a medium (f^2^=0.15) effect size for consent conditions, under alpha=.05. This power calculation, which assumed a single observation per participant, is conservative as the actual study will have 2 observations per participant (1 for each simulated consent process). Therefore, the actual study will have a larger statistical power to detect an effect size of 0.084 for the consent condition. As the test of consent condition effect sizes based on the parental WTP scores is a within-participant comparison rather than an across-participant comparison, a test of the parental consent condition effect size will have at least 80% power to detect an even smaller effect size compared with tests for consent condition effect sizes using the adolescent WTP scores. We project the total enrollment of 264 participants; however, as this is a multi-site project with simultaneous recruitment of a hard-to-reach population, we may schedule and enroll more subjects than anticipated.

### Measures

#### Quantitative Measures

In addition to the primary outcomes, the CASI includes questions that measure covariates of interest, including socioeconomic status, gender identity and sexual orientation, degree of parental monitoring, extent of worry about HIV infection, and medical mistrust. The measures are summarized in Table 1.

**Table 1:** Consent 2.0 quantitative measures.

Content	Scale (administered to adolescents, parents, or both)	Description of items included
Demographics	ATN^a^ data harmonization guidelines [33]	Age, race or ethnicity, sexual orientation, gender identity, education, employment, health insurance status, city, living situation
Socioeconomic status	FAS-III^b^ (adolescents) [34,35]	Seven questions adapted from the FAS-III, measuring a family’s financial status based off of the number of vehicles, computers, bathrooms; adolescents having their own bedroom; if the family has a dishwasher; the number of times the family traveled outside the United States; and overall perception of the family’s financial status
Social support	MSPSS^c^—modified (adolescents) [36]	Four Likert questions on a 7-point scale ranging from very strongly disagree to very strongly agree, measuring parental support and relationships with adolescents
Parental monitoring	Parental monitoring scale—modified (adolescents and parents) [37]	Twenty-five statements on a 5-point scale ranging from strongly disagree to strongly agree, measuring parental knowledge, disclosure, solicitation, and control. Adolescent statements such as “My parent(s) know what I do during my free time.” Parent statements such as “I know what my teen does during their free time.”
Medical mistrust	The group-based medical mistrust scale—modified (adolescents and parents) [38]	Six 5-point Likert items that measure the degree to which the participant trusts medical researchers
Communication	Communication with parents (adolescents and parents) [39]	Five questions asking the number of times parents and adolescents have communicated about relationships, sex, sexually transmitted infections (HPV^d^ and HIV), same-sex relationships, and using a condom. Answers range from Never, Once/twice, Many times, and Don’t know
Concern about HIV	HIV risk perception (adolescents and parents) [40]	Two 5-point Likert questions about adolescent worry of being infected with HIV/AIDS and parent worry of their adolescent being infected with HIV/AIDS
Sexual behavior	Sexual behavior (adolescents) [41]	Five questions for adolescents regarding sexual intercourse partners

^a^ATN: Adolescent Medicine Trials Network for HIV/AIDS Interventions.

^b^FAS-III: family affluence scale-III.

^c^MSPSS: multidimensional scale of perceived social support.

^d^HPV: human papillomavirus.

#### Qualitative Measures

The debriefing interview is designed to explore adolescent and parent perspectives on the various consent conditions in greater depth and to better understand the role of study features, family, and adolescent characteristics in WTP or support the hypothetical research studies. At the start of the interview, the participants are informed that the interview will be recorded and transcribed and are asked to select a pseudonym for the researchers to use. The adolescent debriefing interview consists of 5 sections: (1) general opinions about participating in HIV prevention studies, (2) opinions on the 2 specific studies, (3) relationship with parents, (4) opinions about parental involvement in the consent process, and (5) options and opinions for consenting to future studies. The parent debriefing interview consists of 4 sections: (1) general opinions about HIV prevention studies, (2) relationship with their teenager, (3) opinions about parental involvement in the consent process, and (4) options and opinions for consenting in future studies.

### Data Collection Methods

#### Screening

Adolescents and parents of adolescents are screened either online or in-person using CASIs developed in Qualtrics. Qualtrics is a Web-based system appropriate for use with sensitive data, including those data protected by the Health Insurance Portability and Accountability Act of 1996. Data are stored on secure servers and protected by firewalls. Information regarding their eligibility for the study will be collected, along with the first 5 digits of their street address and first 4 characters of their apartment or unit, where applicable, which together create a 9-digit household ID code. At the time of enrollment, an eligible participant’s household ID code is checked for matches to previously enrolled participants to ensure an independent sample.

#### Study Visit

Participants complete the CASI within the Qualtrics system, using iPad tablets (Apple Inc) provided by the study. No data are stored on the iPad tablet’s hard drive. The entire study visit, including UBACCs and debriefing interviews, is digitally audio-recorded with participant consent. Immediately upon completion, the digital audio files are uploaded to a secure server in Indiana University (IU). The audio recordings are transcribed verbatim. Transcripts are checked against audio recordings for accuracy.

Data regarding study completion or early termination and protocol deviations are collected using case report forms developed within Qualtrics. These reports remain confidential; no personal identifying information is collected.

### Analysis Plan

#### Quantitative Analysis

All analyses will be performed separately for adolescents and parents. Analysis plans for each set of participants are described in the following sections.

#### Analysis of Primary Adolescent Outcomes

Total WTP (sum of scores for both ATN 113 and HPTN 077) will be assessed by a linear model with the categorical consent condition acting as an independent term. Cohen effect size will be calculated from the model’s R^2^ as R^2^/(1-R^2^). To obtain 95% CIs**,** 2000 bootstrap samples will be generated by resampling the data with a replacement, fitting the model, and obtaining the R^2^ for each sample. We will then calculate the aforementioned Cohen effect size for each bootstrap sample and select the 2.5th and 97.5th percentiles of the bootstrap distribution to calculate the corresponding 95% CIs.

Adolescents’ scores of WTP for each study will be modeled by a repeated measures model with independent variables of (1) the consent condition and (2) the study and an exchangeable covariance structure. The covariance structure incorporates into the model the potential correlation of observations from the same subject. The model will be fit using generalized estimating equation methodology. R^2^_marg_ and Cohen effect size will be calculated [42]. A bootstrap algorithm similar to the aforementioned description will be used to obtain the 95% CIs of the effect size.

#### Analysis of Primary Parent Outcomes

Parent acceptability scores for each of the vignettes and studies will be assessed by a linear model with an independent term of consent condition, similar to the analysis of the adolescent outcomes. Cohen effect size will be calculated from the model’s R^2^ as R^2^/(1-R^2^). To obtain 95% CIs**,** 2000 bootstrap samples will be generated by resampling the data with a replacement, fitting the model, and obtaining the R^2^ for each sample. We will calculate the aforementioned Cohen effect size for each bootstrap sample and select the 2.5th and 97.5th percentiles of the bootstrap distribution to calculate the corresponding 95% CIs.

#### Model for Primary Outcomes

Adolescents’ scores of WTP for each study will be modeled by a repeated measures model with independent variables of (1) the consent condition and (2) the study and an exchangeable covariance structure. The covariance structure incorporates into the model the potential correlation of observations from the same subject. The model will be fit using generalized estimating equation methodology; R^2^_marg_ and Cohen effect size will be calculated [42]. A bootstrap algorithm similar to the aforementioned description will be used to obtain the 95% CIs of the effect size. For both adolescents and parent outcomes, aside from the consent condition and trial type, the models will also account for the 2 stratification variables: adolescent’s sex assigned at birth and the study site.

Heterogeneity of consent condition and trial type effects across study sites will be evaluated under the closed testing procedure paradigm [43,44]. First, we will conduct a single overall hypothesis test at the level alpha=.05 for testing the null hypothesis that there is no interaction between consent condition and trial type effects by study site. If this test is statistically significant, we will consider separate interaction tests for the consent condition and trial type. If any of these tests are statistically significant, site-specific estimates of the corresponding effects will be presented to supplement the preplanned analyses.

We will also evaluate if race, ethnicity, outness, concern about HIV, family context (frequency of communication and parental monitoring), medical mistrust, and other demographic and socioeconomic factors affect the primary outcomes (adolescents’ hypothetical WTP and parents’ perceptions of acceptability of consent methods). We will examine these effects by adding variables into the models to determine if they moderate the relationship between the consent condition and WTP (adolescents) and acceptability (parents) scores.

The primary outcome analyses will be based on the observed data. Type B multiple imputation [45] will be used to address the missing data instead of the traditional Rubin type A multiple imputation. This is because the latter method, unlike type B multiple imputation, produces biased standard error estimates and *P* values if there are auxiliary variables in the imputation models owing to model uncongeniality [46,47]. The use of auxiliary variables can make the missing at random assumption more plausible in practice [48,49].

#### Qualitative Analysis

**Figure 1:**
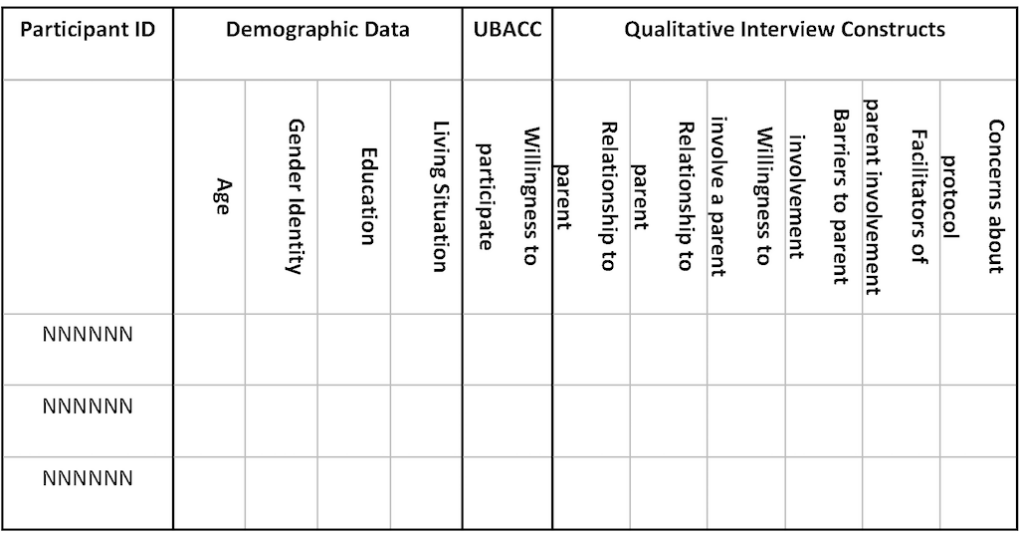
A sample qualitative data analysis matrix for adolescent participants. UBACC: University of California, San Diego Brief Assessment of Capacity to Consent.

Our analytic approach to the qualitative data collected during debriefing interviews is a qualitative description, as described by Sandelowski [50]. Qualitative descriptive methods provide an in-depth description of experiences shared by a group facing a common challenge and are particularly useful for generating summaries of information to guide future interventions. The qualitative analysis team will analyze the transcripts using conventional content analysis techniques as described by Hsieh and Shannon [51]. Using QSR International’s NVivo version 12 textual analysis software, each text unit (eg, meaningful phrase, sentence, or story relevant to the study aims) will be coded with a short phrase that reflects its essence. A case-ordered meta-matrix [52] will be constructed, with each row representing an individual case and each column representing selected variables drawn from quantitative measures (eg, sexual orientation and gender identity) or salient constructs derived from the interviews (eg, relationship with parent/teen). For ease of comparison, separate matrices will be made for adolescents and parents. The research team will categorize all the codes in each column and provide a description of each category to describe the variable fully from the parents’ and adolescents’ perspectives. For example, all the codes under barriers to parental involvement will be categorized to provide a list of barriers, and the barriers identified by each group will be compared with considerable differences in the 2 groups’ perspectives. An example data matrix is shown in Figure 1.

### Human Subjects

This protocol was reviewed and approved by the IRB at IU, which served as the single IRB by a reliance agreement between it and the IRBs affiliated with our research sites. After a screened participant is determined to be eligible, she or he receives a study information sheet. The study purpose and procedures are discussed, and all questions are answered during the informed consent process. Verbal informed consent is obtained before any study-related procedures are performed. Minors do not require parental consent to participate. Enrollment occurs after participant consent is obtained.

### Monitoring

#### Study Monitoring

Implementation of the study is monitored by the study team review committee (STRC), which includes the protocol chair, Eunice Kennedy Shriver National Institute of Child Health and Human Development (NICHD) health science administrator, 1 coinvestigator, the Consent 2.0 program manager, and the IU data manager. The STRC meets at least monthly. During these meetings, the STRC reviews enrollment reports, reports on early discontinuation of the 1-day study visit, and reports on adverse events (AEs) that have occurred since the last STRC meeting or previously reported AEs for which new information is available. All AEs are reviewed within one week of occurrence; if the next scheduled STRC meeting is more than 7 days after an AE occurred, the team will convene a special meeting to address it.

#### Data Monitoring

Data monitoring is conducted on a weekly basis by the IU data manager to detect any issues that require reporting or correction. If any data corrections are necessary, the data manager will contact the PI to discuss and review any necessary corrections. A query notifications system is used to track any protocol deviations or problems that arise during study visits. Preliminary analyses will be conducted to detect potential errors in the data collection process and to assess the adequacy of planned enrollment.

## Results

Funding for this study began in late 2016. Initial IRB approval was secured in July of 2017. All recruitment, enrollment, and data collection occurred between April 2018 and September 2019. The study enrolled 131 adolescents and 125 parents. As of January 2020, analysis is underway with a primary results manuscript anticipated to be published by mid-2020.

## Discussion

### Strengths and Limitations

Adolescents—especially adolescents of color—are disproportionately affected by HIV but underrepresented in biomedical HIV prevention trials that may benefit them as individuals and as a collective. The low engagement of adolescents in biomedical research creates delays in their access to new prevention tools and subsequently contributes to HIV disparities in this age group. Researchers and policy makers have called for the inclusion of adolescents in biomedical research as a matter of justice [4,6,7,53,54]. However, there are limited empirical data regarding the consent-related needs and preferences of minor adolescents at a high risk of HIV acquisition and limited data about parents’ perspectives on the issue. This project responds to both the identified disparities in minors’ access to clinical trials and the limited empirical data available for creating resolutions to the problem that are acceptable for both adolescents and parents. Furthermore, the project specifically explores the intersection of race, ethnicity, sexual orientation, gender identity, and medical mistrust as they relate to ethical concerns about engaging minors in biomedical HIV prevention research studies.

There are several anticipated limitations of this study. First, we are enrolling a relatively small sample size of approximately 144 adolescents and 120 parents. The sample comes from 4 geographic regions in the United States and is recruited through a variety of methods to ensure socioeconomic, racial, ethnic, and sexual diversity, but it cannot be considered representative of all relevant stakeholders. A second limitation is the hypothetical nature of the study. We made every effort to simulate a real clinical trial—we are using risk criteria from real trials, we are recruiting from sites that participate in trials similar to those that we selected for the study, and we are simulating the consent process by acting just as though the participant is actually enrolling in the trial. Nevertheless, a participant’s hypothetical choice may be different from the choice they make in reality. A third limitation is the possibility of sampling bias. Participants in this study are volunteers, many of whom self-refer to the study. Thus, they may be more likely to participate in medical research or more open to the idea, generally. A random community sample may produce different results than this volunteer sample. Finally, our sample size calculation has not accounted for the possibility of unbalanced covariates owing to chance. If we need to adjust the model to account for unbalanced covariates, this will result in a reduction in the effect size detectable with 80% power.

### Conclusions

We anticipate the results of this project will be useful to research networks, principal investigators, policy makers, and regulatory bodies who must make decisions about inclusion and exclusion criteria and consent requirements for research participants.

## Abbreviations

AE: adverse event
ATN: Adolescent Medicine Trials Network for HIV/AIDS Interventions
ATN 113: Adolescent Medicine Trials Network for HIV/AIDS Interventions protocol 113
CASI: computer-assisted self-interview
HPTN 077: HIV Prevention Trials Network protocol 077
IRB: institutional review board
IU: Indiana University
MTN: Microbicide Trials Network
NICHD: Eunice Kennedy Shriver National Institute of Child Health & Human Development
NIH: National Institutes of Health
PrEP: preexposure prophylaxis
STRC: study team review committee
TDF-FTC: tenofovir disoproxil fumarate and emtricitabine
UBACC: University of California, San Diego Brief Assessment of Capacity to Consent
WTP: willingness to participate
